# Supplementary material for: Exposure to a Low-Oxygen Environment Causes Implantation Failure and Transcriptomic Shifts in Mouse Uteruses and Ovaries
Source: Biomedicines. 2024 May 5;12(5):1016. doi: 10.3390/biomedicines12051016 (PMC11118081; doi:10.3390/biomedicines12051016)
Supplement: Supplementary file 1 [file biomedicines-12-01016-s001.zip › Supplementary Figures S1-S2.pdf]

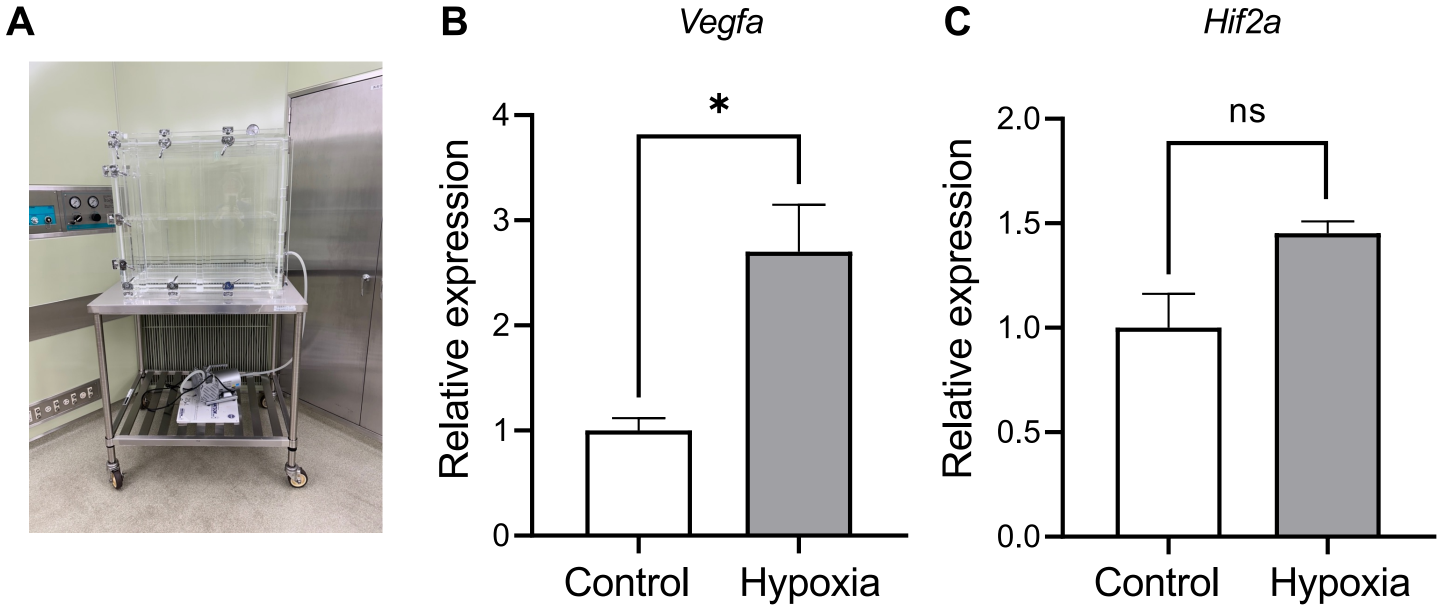

Figure S1: (**A**) Arrangements of the chamber and the pump. (**B**) *Vegfa* and (**C**) *Hif-2α* transcript levels in control and hypoxic groups of mouse uteruses at GD1.5. * $p < 0.05$, ns: not significant.

A

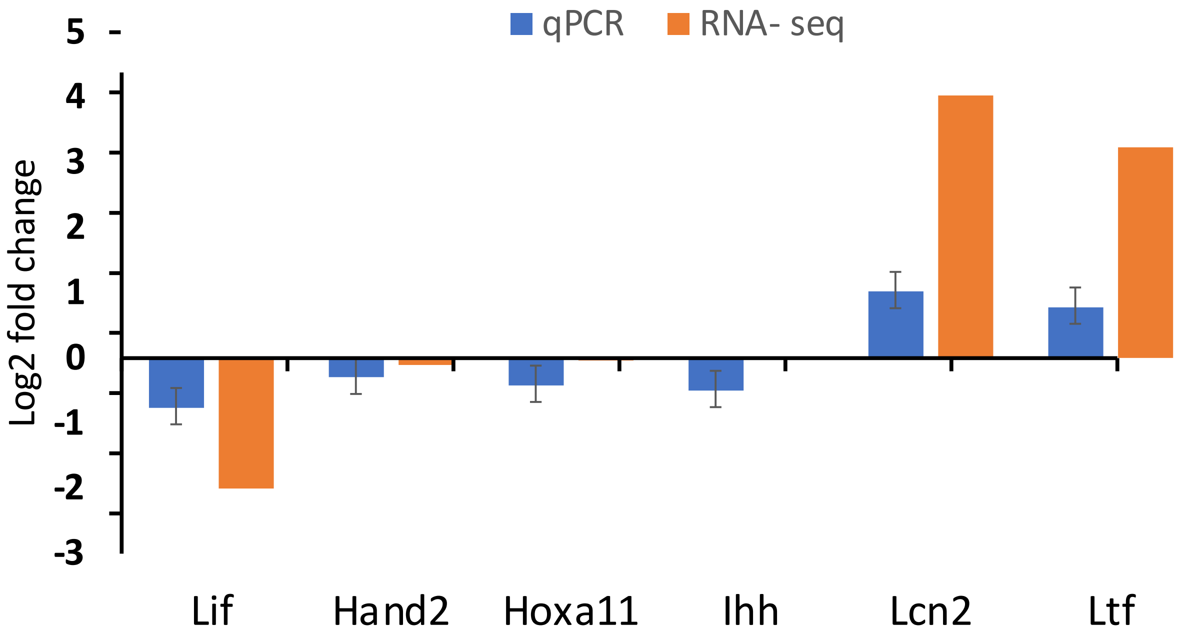

B

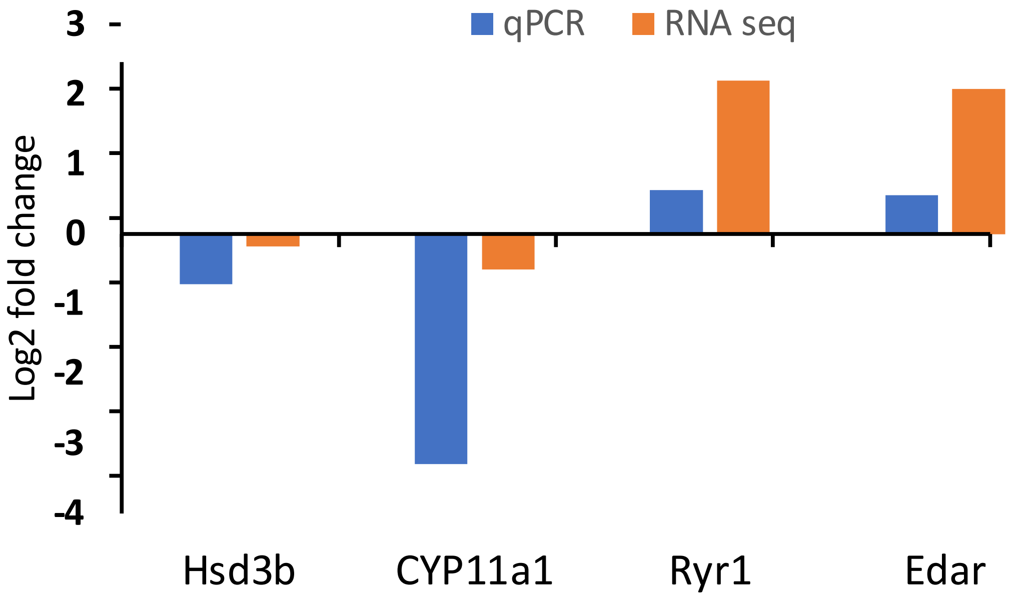

Figure S2: Validation of RNA-seq data by quantitative PCR (qPCR). (A) Uterus, (B) Ovary.
